# Doxazosin Stimulates Galectin-3 Expression and Collagen Synthesis in HL-1 Cardiomyocytes Independent of Protein Kinase C Pathway

**DOI:** 10.3389/fphar.2016.00495

**Published:** 2016-12-20

**Authors:** Xiaoqian Qian, Mingyang Li, Mary B. Wagner, Guangping Chen, Xiang Song

**Affiliations:** ^1^Cardiovascular Center, The Fourth Affiliated Hospital, Harbin Medical UniversityHarbin, China; ^2^Department of Physiology, Emory University, AtlantaGA, USA; ^3^Heart Research and Outcomes Center, Children’s Healthcare of Atlanta and Department of Pediatrics, Emory University School of Medicine, AtlantaGA, USA

**Keywords:** adrenergic receptor, protein kinase C, cardiac fibrosis, collagen

## Abstract

Doxazosin, a drug commonly prescribed for hypertension and prostate disease, increases heart failure risk. However, the underlying mechanism remains unclear. Galectin-3 is an important mediator that plays a pathogenic role in cardiac hypertrophy and heart failure. In the present study, we investigated whether doxazosin could stimulate galectin-3 expression and collagen synthesis in cultured HL-1 cardiomyocytes. We found that doxazosin dose-dependently induced galectin-3 protein expression, with a statistically significant increase in expression with a dose as low as 0.01 μM. Doxazosin upregulated collagen I and α-smooth muscle actin (α-SMA) protein levels and also induced apoptotic protein caspase-3 in HL-1 cardiomyocytes. Although we previously reported that activation of protein kinase C (PKC) stimulates galectin-3 expression, blocking the PKC pathway with the PKC inhibitor chelerythrine did not prevent doxazosin-induced galectin-3 and collagen expression. Consistently, doxazosin treatment did not alter total and phosphorylated PKC. These results suggest that doxazosin-stimulated galectin-3 is independent of PKC pathway. To determine if the α1-adrenergic pathway is involved, we pretreated the cells with the irreversible α-adrenergic receptor blocker phenoxybenzamine and found that doxazosin-stimulated galectin-3 and collagen expression was similar to controls, suggesting that doxazosin acts independently of α1-adrenergic receptor blockade. Collectively, we show a novel effect of doxazosin on cardiomycytes by stimulating heart fibrosis factor galectin-3 expression. The mechanism of action of doxazosin is not mediated through either activation of the PKC pathway or antagonism of α1-adrenergic receptors.

## Introduction

Doxazosin, a piperazinyl quinazoline compound, is a long lasting selective inhibitor of α1-adrenergic receptors. It is a commonly prescribed treatment for patients with hypertension (Black, 2003; Ceral and Solar, 2009) and enlarged prostate (Clarke, 1998). Doxazosin effectively lowers blood pressure by blocking norepinephrine binding to α1-adrenergic receptors, relaxing smooth muscle cells, subsequently decreasing vascular tone and reducing peripheral vascular resistance. Doxazosin is also used to treat urinary retention problems in patients with benign prostatic hyperplasia. By blocking the action of α1-adrenergic receptors, doxazosin treatment leads to relaxation of the smooth muscles surrounding the prostate, which ease urine flow and decrease bladder outlet obstruction (Tahmatzopoulos et al., 2004). In addition, doxazosin has shown a beneficial effect on lipids by modestly lowering both total cholesterol and triglycerides (Remaley, 2007).

The utility of doxazosin for the treatment of hypertension was called into question in March of 2000. A large national prospective study ALLHAT (the Antihypertensive and Lipid-Lowering Treatment to Prevent Heart Attack Trial) sponsored by the National Heart, Lung, and Blood Institute (NHLBI) and conducted from 1994 to 2002 showed that compared to the diuretic chlorthalidone, doxazosin was associated with an increased risk of cardiovascular events, particularly congestive heart failure (ALLHAT Collaborative Research Group, 2000). Specifically, the study showed that the risk of congestive heart failure was twice as high in the doxazosin group compared to patients treated with the chlorthalidone. Thus, the doxazosin-related portion of the study was discontinued. The increased risk of heart failure caused by doxazosin might be due to the blocking of specific receptors in heart muscle cells. Additional studies have shown that doxazosin causes apoptosis in cardiomyocytes and can arrest the cell cycle of human coronary smooth muscle cells. However, these effects are independent of α1-adrenergic receptors (Hu et al., 1998; Gonzalez-Juanatey et al., 2003; O’Connell et al., 2006), indicating that doxazosin’s ability to cause heart failure requires mechanisms that extend beyond α1-adrenergic receptors blockade.

Galectin-3 is implicated in heart failure. This was first reported by Sharma et al., who identified galectin-3 as the strongest predictor of heart failure among 48 genes that were upregulated in failing hearts compared to hearts with compensated hypertrophy (Sharma et al., 2004). Several studies have shown that galectin-3 is up-regulated in cardiac remodeling and is a mediator of cardiac fibrosis (Sharma et al., 2004; Liu et al., 2009; Yu et al., 2013). Galectin-3 knockout mice showed decreased fibrosis and preserved cardiac function in response to heart failure induction compared to controls (Yu et al., 2013). In a clinical study of patients with heart failure, increased circulating levels of galectin-3, used as a marker of cardiac fibrosis, were associated with an increased risk for incident heart failure (Ho et al., 2012).

We recently reported that activation of the PKC pathway increases galectin-3 expression in cultured cardiomyocyte HL-1 cells. Moreover, we also showed that PKC-α promotes cardiac fibrosis and heart failure by stimulation of galectin-3 expression (Song et al., 2015). In the present study, we follow-up on the findings of the ALLHAT trial by investigating the mechanisms by which doxazosin used as an anti-hypertensive is associated with an increase in clinical heart failure. We performed a series of *in vitro* experiments to explore whether galectin-3 is involved in the action of doxazosin on cardiomyocytes. We found that doxazosin stimulated galectin-3 expression and collagen synthesis in HL-1 cardiomyocytes. In addition, we found that this effect did not require adrenergic receptor activity and was not mediated by the PKC pathway.

## Materials and Methods

### Cardiomyocyte Culture

HL-1 cardiomyocytes, kindly provided by Dr. Claycomb from Louisiana State University Medical Center, were cultured in an optimized Claycomb medium as previously described (Song et al., 2015). Claycomb medium (Sigma, 51800C) was supplemented with 10% fetal bovine serum (Sigma, F2442), 4 mM L-glutamine, 0.1 mM norepinephrine (Sigma, A0937) and 100 U/ml penicillin and 100 μg/ml streptomycin. Cells were grown in culture flasks, dishes or plates pre-coated with 5 μg/ml fibronectin (Sigma, F1141) at 37°C in a humidified atmosphere of 95% air and 5% CO_2_.

### Chemicals and Cell Treatment

For pharmacologic cell treatment, HL-1 cells were grown to confluence and serum starved for 12 h. The cells were then treated with 0.01∼10 μM doxazosin (Sigma, D9815), 0.1 μM prazosin (Sigma, P7791), 2 μM PKC inhibitor chelerythrine (Sigma, C2932), or 1 μM phenoxybenzamine (Sigma, B019) for the indicated time. Doxazosin was prepared as a 10 mM stock solution in DMSO and stored at -20°C.

### Western Blot Analysis

After treatment, cells were lysed with an ice-cold modified radio-immunoprecipitation assay (RIPA) buffer (150 mM NaCl, 10 mM Tris-HCl, pH 7.5, 1 mM EDTA, 1% Triton X-100, 1% sodium deoxycholate, 0.1% SDS, and protease inhibitors). Cellular lysates were then centrifuged for 10 min at 10,000 rpm and the supernatant was collected. Protein concentration was determined by the Bradford method using BioRad protein assay. Equal amounts of proteins (50∼100 μg/lane) were loaded and separated by SDS-PAGE gels and transferred to nitrocellulose membranes (Bio-Rad). After blocking with 5% non-fat milk in phosphate buffered saline tween-20 (PBST), membranes were incubated with primary antibodies overnight at 4°C, followed by horseradish peroxidase (HRP)-conjugated secondary antibody. The protein abundance was detected using enhanced chemiluminescence ECL system (Amersham Biosciences). Band density was quantified using ImageJ software (National Institutes of Health). The results were expressed as percentage of the control group. The following antibodies were used in this study: galectin-3 hybridoma (TIB-166; ATCC), Col Iα1 (sc-8784; Santa Cruz), α-SMA (A2547; Sigma), PKC-α (sc-8393; Santa Cruz), PKC-α (phospho Thr497) (GTX61959; GeneTex), PKC-α (phospho Thr638/641) (9375; Cell signaling), GAPDH (sc-25778; Santa Cruz), HRP anti-rabbit IgG (NA934; Fisher), and HRP-goat anti-mouse IgG (115-036-062; Jackson Immuno Research).

### Data Analysis and Statistics

The protein levels quantified by densitometry were expressed as mean ± SD. The statistically significant differences were assessed by ANOVA with *post hoc* Tukey HSD test.

## Results

### Doxazosin Stimulates Galectin-3 Expression in Cardiomyocytes

**FIGURE 1:**
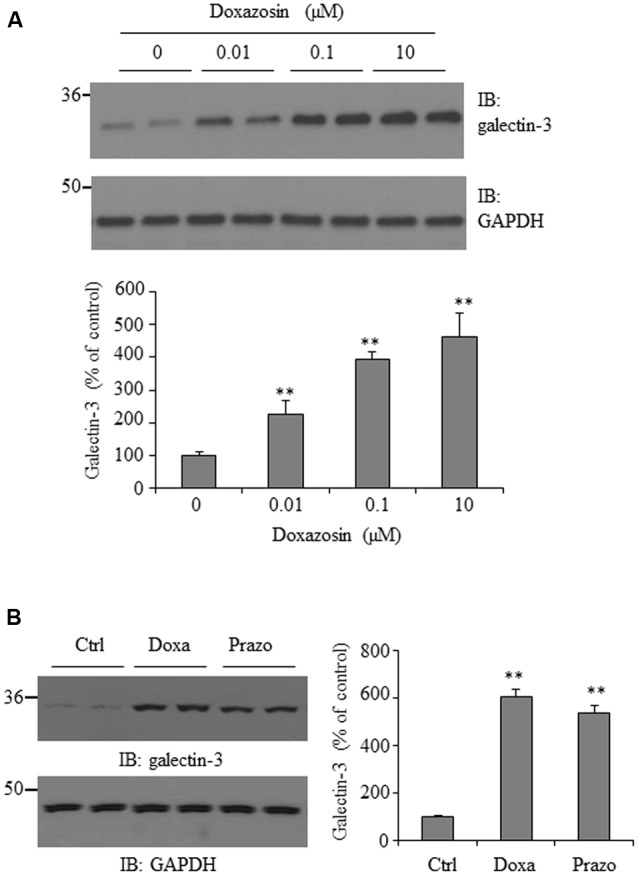
**Effect of doxazosin on galectin-3 expression. (A)** HL-1 cardiomyocytes were treated with doxazosin at doses of 0, 0.01, 0.1, and 10 μM for 12 h. **(B)** HL-1 cells were incubated with 0.1 μM doxazosin (Doxa) or 0.1 μM prazosin (Prazo) for 12 h. Cells were collected and galectin-3 expression was analyzed by Western blot. Experiments were performed in triplicate and data is shown as the mean ± SD (*n* = 3, ^∗∗^*p* < 0.01 compared to control).

Galectin-3 is thought to enhance fibrosis, a central process in both maladaptive cardiac remodeling and heart failure (Sharma et al., 2004). To explore whether the antihypertension drug doxazosin could influence galectin-3 expression, HL-1 cardiomyocytes were treated with doxazosin at different doses for 12 h, and galectin-3 expression was measured by Western blot. As shown in **Figure 1A**, doxazosin stimulated galectin-3 protein expression in a dose-dependent manner, with an initial increase seen at a low concentration of just 0.01 μM. In patients, doxazosin plasma levels of 122 nM are recorded at the therapeutic dose of 8 mg (Frick et al., 1986). In addition, we examined whether another quinazoline based alpha1 antagonist prazosin, similarly, stimulates the expression of galectin-3. As shown in **Figure 1B**, prazosin also stimulated galectin-3 protein expression in HL-1 cardiomyocytes.

### Effect of Doxazosin on Collagen I and α-SMA Expression

**FIGURE 2:**
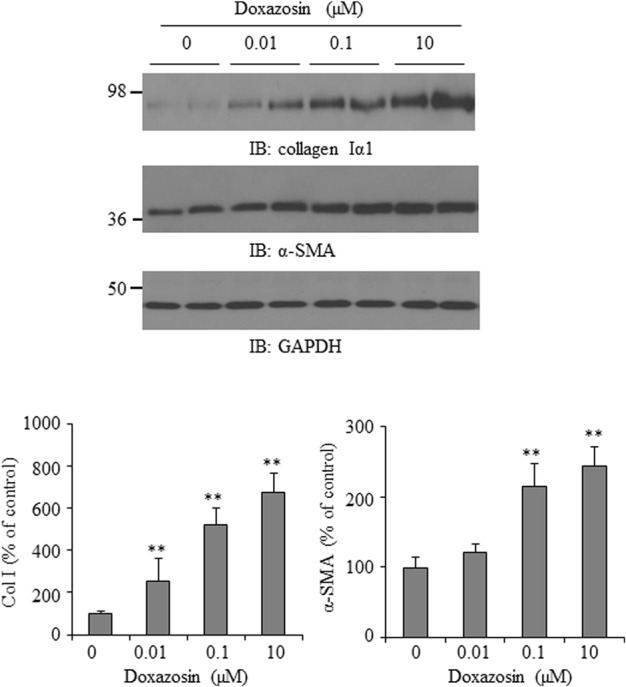
**Effect of doxazosin on collagen I and α-SMA expression.** HL-1 cardiomyocytes were treated with doxazosin at increasing doses for 12 h. Collagen Iα1 and α-SMA expression was analyzed by Western blot and data is shown as the mean ± SD (*n* = 3, ^∗∗^*p* < 0.01 compared to control).

Increased interstitial and perivascular fibrosis are characteristic of cardiomyopathy (O’Connell et al., 2006). Collagen I is one of the major extracellular matrix proteins accumulated in cardiac fibrosis. Additionally, the alpha-smooth muscle actin (α-SMA) isoform is normally expressed in differentiating cardiomyocytes and is a marker for myocardial hypertrophy in adult hearts (Kern et al., 2013). We evaluated the effect of doxazosin on collagen Iα1 and α-SMA expression in HL-1 cardiomyocytes. In **Figure 2**, treatment of HL-1 cells with doxazosin for 12 h stimulated collagen I α1 and α-SMA expression.

### Doxazosin-Induced Collagen 1 and Galectin-3 Was Not Affected by PKC Blockade

**FIGURE 3:**
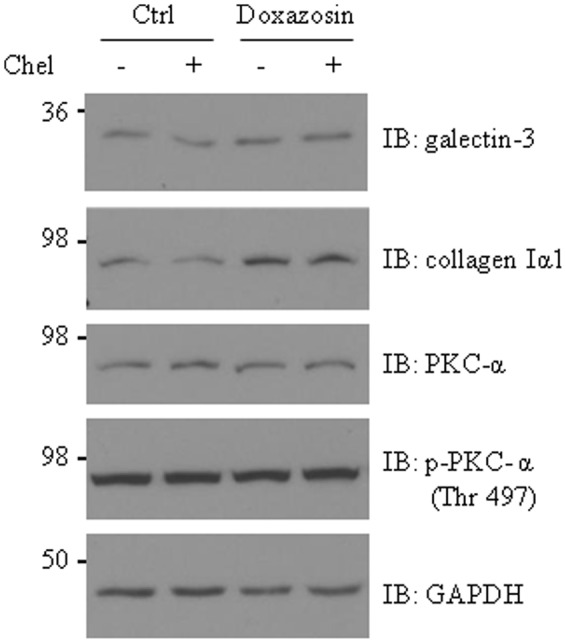
**Protein kinase C (PKC) is not involved in doxazosin-stimulated collagen and galectin-3 expression.** HL-1 cardiomyocytes were pre-treated with 2 μM chelerythrine (Chel) for 30 min then treated with 1 μM doxazosin for 12 h. Galectin-3, collagen I, and PKC were assessed by Western blot with appropriate antibodies.

Activation of PKC pathway promotes galectin-3 expression and cardiac fibrosis in HL-1 cardiomyocytes and in heart failure hearts (Song et al., 2015). Thus, we investigated whether PKC is involved in mediating doxazosin-induced galectin-3 and collagen expression. HL-1 cells were pre-treated with PKC inhibitor chelerythrine (Chel) for 30 min prior to doxazosin treatment. Inhibition of PKC did not block doxazosin-induced galectin-3 and collagen expression (**Figure 3**).

### Doxazosin Does Not Change PKC Protein Expression

**FIGURE 4:**
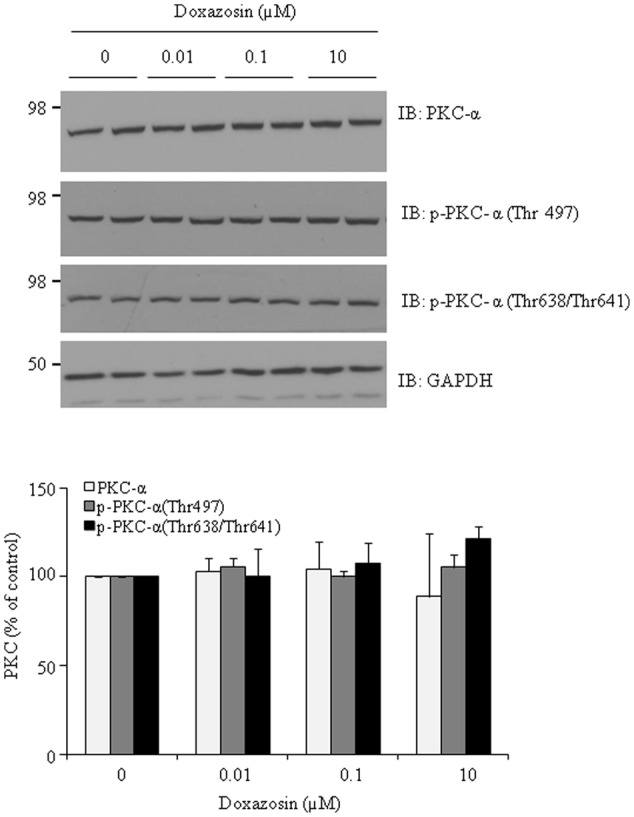
**Effect of doxazosin on PKC protein expression.** HL-1 cardiomyocytes were treated with 1 μM doxazosin for 12 h, total PKC-α protein and phosphorylated PKC-α at Thr497, Thr638 and Thr641 were analyzed by immunoblotting with appropriate antibodies. Experiments were performed in triplicate and data is shown as the mean ± SD. Statistics show no significance of doxazosin treated groups compared to control.

Both clinical and experimental animal studies reveal the association of PKC activation with cardiac hypertrophy and heart failure (Bowling et al., 1999; Bayer et al., 2003). To further investigate whether doxazosin affects the PKC pathway, expression of total PKC-α was evaluated after doxazosin treatment. As presented in **Figure 4**, doxazosin did not change total PKC-α protein expression. By utilizing antibodies specific for phosphorylated PKC-α, we assessed whether activated PKC-α was altered even without changes in total PKC-α. However, levels of phosphorylated PKC-α at Thr497, Thr638, and Thr641 were not changed by doxazosin treatment.

### Doxazosin Stimulates Apoptotic Protein Caspase-3 Expression in HL-1 Cardiomyocytes

**FIGURE 5:**
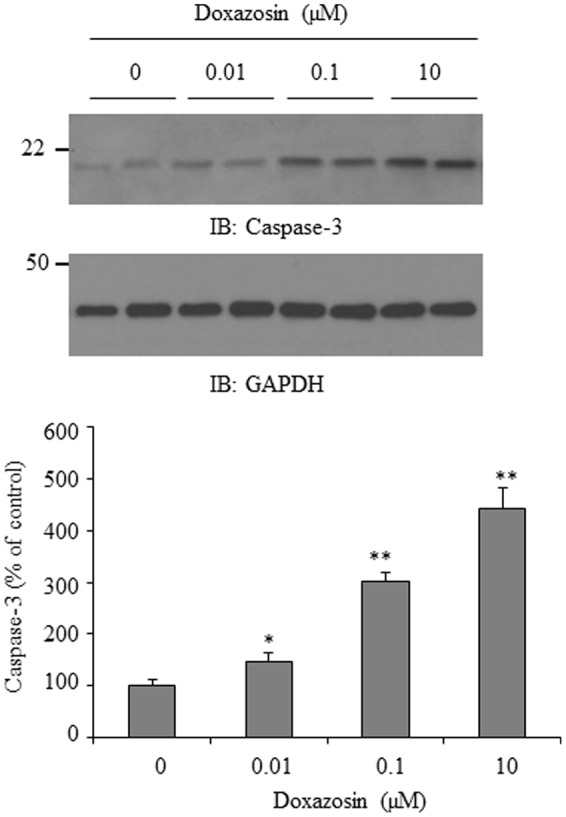
**Doxazosin induces cell apoptosis in HL-1 cardiomyocytes.** HL-1 cells were treated with doxazosin at increasing concentrations for 12 h, and the apoptotic protein caspase-3 was examined by Western blot. Caspase-3 signals were quantified and is shown as the mean ± SD (^∗^*p* < 0.05, ^∗∗^*p* < 0.01 compared to control, *n* = 3).

Apoptosis is recognized as a cause of interstitial fibrosis in other organs (O’Connell et al., 2006). Doxazosin induces cell apoptosis in prostate cancer cells (Garrison and Kyprianou, 2006; Forbes et al., 2016) as well as in cardiomyocytes (Gonzalez-Juanatey et al., 2003). Caspase-3 is a critical apoptotic molecule, as it is responsible for the proteolytic cleavage of various regulatory proteins, such as poly (ADP-ribose) polymerase (PARP). We examined the expression of caspase-3 in HL-1 cardiomyocytes in response to doxazosin treatment. As expected, doxazosin increased caspase-3 protein levels (**Figure 5**), suggesting that doxazosin similarly increases cellular apoptosis.

### Doxazosin Stimulated Galectin-3 and Collagen Expression Does Not Involve α1-Adrenergic Receptor Activity

**FIGURE 6:**
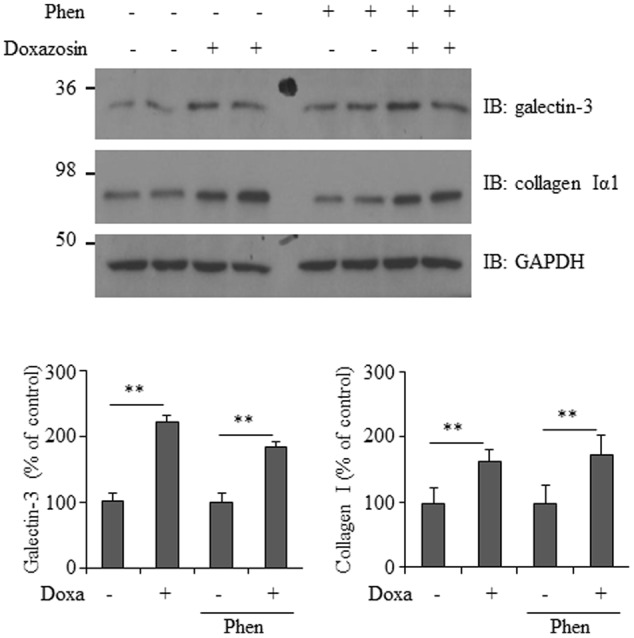
**Effect of α1-adrenergic receptor blocker on doxazosin-stimulated galectin-3 and collagen expression.** HL-1 cells were pre-treated with phenoxybenzamine (Phen) for 4 h then treated with doxazosin (Doxa) for 12 h. Galectin-3 and collagen I were analyzed by Western blot. Signals were quantified as the mean ± SD (^∗∗^*p* < 0.01 compared to control without Doxa treatment, *n* = 3).

Doxazosin is a selective α1-adrenergic receptor blocker that inhibits the binding of norepinephrine to the receptor on the cell membrane. To determine if α1-adrenergic receptor activity is required for doxazosin-induced increase in galectin-3 and collagen, we utilized phenoxybenzamine, an irreversible α-adrenergic blocker, which permanently binds to α-adrenergic receptors. HL-1 cells were pre-treated with phenoxybenzamine at a concentration of 1 μM for 4 h, followed by doxazosin treatment. Blocking α-adrenergic receptors did not inhibit doxazosin-induced galectin-3 and collagen expression (**Figure 6**).

## Discussion

Galectin-3 has been linked to interstitial cardiac fibrosis and is a purported prognostic marker in patients with heart failure (Sharma et al., 2004; Yu et al., 2013; Song et al., 2015). In the present study, we found that doxazosin treatment stimulated galectin-3 expression and collagen synthesis in cultured HL-1 cardiomyocytes, suggesting a possible new mechanism of doxazosin caused heart injury by stimulating heart fibrosis factor galectin-3 expression.

Protein kinase C (PKC) is a group of serine/threonine kinases involving many physiological and pathological processes. Animal and human studies have revealed that activation of PKC-α or an increase in PKC-α expression is associated with heart failure (Braz et al., 2004; Liu and Molkentin, 2011). Inhibition of PKC by ruboxistaurin attenuated diastolic dysfunction, myocyte hypertrophy, collagen deposition, and preserved cardiac contractility in a rat diabetic heart failure model (Connelly et al., 2009). We recently reported that activation of the PKC pathway increased galectin-3 protein expression (Song et al., 2015). This prompted us to examine whether PKC is the downstream target of doxazosin and mediates doxazosin stimulated galectin-3 expression. However, our data did not support an involvement of PKC in doxazosin-induced galectin-3 expression and collagen synthesis. Neither did doxazosin treatment alter the expression of phosphorylated and total PKC-α.

Although doxazosin is an α1-adrenergic receptor blocker, increasing evidence shows that doxazosin causes many side effects unrelated to its α-adrenergic receptor blocking activity. The anti-cancer activity of doxazosin is largely attributed to apoptotic mechanisms. It has been shown that doxazosin induced apoptosis is via a α1-adrenoceptor–independent pathway (Kyprianou, 2003; Forbes et al., 2016). This was confirmed by an elegant experiment showing doxazosin-induced apoptosis in human embryonic kidney (HEK) cells lacking α-adrenergic receptors (Thomas et al., 2008). In this study, we investigated whether α-adrenergic receptor activity is required for doxazosin stimulated galectin-3 expression. Blockade of α-adrenergic receptors by pre-treatment of cells with phenoxybenzamine did not affect doxazosin induced galectin-3 levels. This is consistent with a study by Ciccarelli et al. (2008) showing that doxazosin stimulation of endothelial cell migration and vascular tube formation does not require α-adrenergic receptor activity and is independent of vasodilatation.

The mechanism of action of doxazosin might be very complicated and is still under investigation. It has been reported that doxazosin can enhance serum-induced extracellular signal-regulated kinase (ERK) activation and phosphorylation of Rb in endothelial cells (Ciccarelli et al., 2008). Garrison et al reported that doxazosin induced apoptosis in prostate cancer cells involves activation of transforming growth factor-β1 (TGF-β1) signaling (Garrison and Kyprianou, 2006). An interesting study performed by Thomas et al. showed that doxazosin only induced apoptosis in hERG-expressing HEK cells, but not in untransfected control HEK cells (Thomas et al., 2008). They propose that the potassium channel hERG serves as the receptor for doxazosin which may contribute to heart failure in patients treated with the antihypertensive drug. By using HL-1 cardiomyocytes, Eiras et al. performed a microarray analysis showing an increase in phosphorylation of p38 MAP kinase and focal adhesion kinase (FAK) in response to doxazosin treatment (Eiras et al., 2006). It will be interesting to know whether p38 MAP kinase and/or FAK signaling pathways are involved in doxazosin stimulated galectin-3 expression and whether the increased heart failure risk by doxazosin is due to activating specific receptors like hERG in heart muscle cells.

## Conclusion

The major finding of this study is that doxazosin caused a dose dependent increase in galectin-3 protein expression in HL-1 cardiomyocytes, a possible new mechanism of doxazosin induced higher incidence of heart failure in the ALLHAT study. We also found that the effects of doxazosin are not dependent on its ability to inhibit the alpha-1 adrenergic receptor as an irreversible antagonist was not able to block this effect of doxazosin. Further studies are needed to elucidate the intracellular signaling pathways mediating doxazosin-induced galectin-3 upregulation in cardiomyocytes.

## Author Contributions

XQ, GC, and XS designed research. XQ and ML performed research. XQ, ML, MW, GC, and XS analyzed data and XQ, MW, GC, and XS wrote the paper.

## Conflict of Interest Statement

The authors declare that the research was conducted in the absence of any commercial or financial relationships that could be construed as a potential conflict of interest.
